# Supplementary material for: Genome-wide gene-environment interaction study uncovers 162 vitamin D status variants using a precise ambient UVB measure
Source: Nat Commun. 2025 Nov 28;16:10774. doi: 10.1038/s41467-025-65820-x (PMC12663108; doi:10.1038/s41467-025-65820-x)
Supplement: Supplementary file 1 — Supplementary Information [file 41467_2025_65820_MOESM1_ESM.pdf]

Genome-wide gene-environment interaction study uncovers 162 vitamin D status variants using a precise ambient UVB measure

Shraim et al., 2025

Supplementary Information

Supplementary Methods 1
Genotype quality control 1
Population Stratification Correction 2
TEMIS data 2
Supplement Intake Covariates 2
High Time Outdoors Group 2
Genetic scores 2
Sensitivity analysis 3
Sample size estimate for replication 3
Replication cohorts 3
Supplementary Tables 4
Table S1: UK Biobank data fields. 4
Table S2: Distribution of raw and standardised log-transformed 25OHD. 4
Table S3. Association of 25OHD with potential covariates 5
Table S4: Association of genetic scores with 25OHD 5
Supplementary sheet tables 5
Supplementary Figures 6
Figure S1: Quality control 6
Figure S2: Distribution of 25OHD 6
Figure S3: Supplement and fish oil intake 7
Figure S4: Distribution of vitamin D and UVB 8
Figure S5: Mirrored Manhattan plot of genome-wide GxE and marginal tests 9
Figure S7: Manhattan plots by CW-D-UVB quintile 11
Figure S8: Manhattan plot of high time outdoors subgroup. 12
Figure S9: Venn diagram of overlap with outdoors subgroup. 13
Figure S10: Distribution of CW-D-UVB in outdoors subgroup 13
Figure S11: Distribution of CW-D-UVB in the European cohort 14
Figure S12: Distribution of genetic scores and association with 25OHD 15
Figure S13: MAGMA tissue expression analysis 16
Figure S14: DAVID functional annotation 17
Figure S15: Manhattan plots by BMI category 18
Figure S16: Venn diagram of overlap across BMI categories 19
Figure S17: Effect estimate and p-value comparison with recent GWAS 20
Figure S18: Manhattan plot of Plink2 GWAS results 21
References 21

Supplementary Methods

Genotype quality control

We quality controlled the imputed genotype data from the UKB in Plink2 **(Fig. S1**). We first filtered the data for imputation quality (MaCH Rsq quality score 0.8 to 2.0). We then filtered for variant and individual missingness >0.02, minor-allele frequency (MAF) >0.01, and Hardy-Weinberg equilibrium p-value > $1x10^{-6}$. Mean heterozygosity and SD were calculated for the White British group and individuals with missing sex or heterozygosity +/- 6SD were excluded. Finally, individuals with mismatched sex were excluded. We discarded relatives based on KING kinship threshold ≥0.0884 (indicating 2nd degree relatives or closer), keeping one participant from each group of relatives.

Population Stratification Correction
After QC, to correct for population stratification in the White British population, principal components were calculated from the LD pruned genetic data with Plink2.0 (--pca). To reduce computational resource use, the flag --approx was also used, as recommended for samples over 50,000.[1] We included the first 10 PCs in the genetic analysis of the White British group.

TEMIS data
The D-UVB data is determined from a parameterisation of D-UVB as a function of satellite-based ozone observations, the solar zenith angle, and the vitamin D action spectrum (as adopted by the International Commission on Illumination[2]), and includes corrections for surface elevation, surface albedo, sun-earth distance and cloudiness. The method is described and validated by Zempila et al.[3]; this has since been upgraded to a higher resolution (see www.temis.nl/uvradiation/product/ for detailed information). The date of blood draw was extracted for each participant. Based on residential location and sample date, an array of daily D-UVB doses was extracted from the TEMIS database. Unlike most other biomarkers, accumulation of vitamin D in the body can be observed in the summer - i.e. times of the high UVB intensity, and diminution in the winter when solar radiation is too weak for vitamin D synthesis to occur. Previous work (Kelly et al. 2016) determined that an UVB-based estimate that most strongly correlates to 25OHD concentration uses data covering a period of 135 days prior to blood sampling, whilst weighting the exposures so that more recent exposures contribute more to the estimate.[4] To weigh UVB dose, we use the recommended half-life of 35 days (which is in accordance with previously reported observations: the half-life of vitamin D in the body has been reported to be about 2 months, while circulating 25OHD has been reported to be broken down after 15 days[5]). Thus, cumulative and weighted UVB (CW-D-UVB) was calculated as $CW\text{-}D\text{-}UVB(x) = \Sigma_{x=1:135}(D\text{-}UVB(x) * e^{-(ln2/y)x}$ (**Eq1**, described in the main **Methods**)."

Supplement Intake Covariates
We adjusted our models for 'vitamin D supplement use' (yes/no) and for 'fish oil supplement use' (yes/no) supplements. The first was based on the 'Vitamin and mineral supplements' self-reported assessment questionnaire. Participants that indicated taking 'Vitamin D' or 'Multivitamins +/- minerals' were assigned *yes*, and all other responses were assigned *no*. Similarly, we used the 'Mineral and other dietary supplements' field, where participants that indicated taking 'Fish oil (including cod liver oil)' were assigned *yes*, and all other responses *no*. Fish oil supplement use was associated with a similar increase in 25OHD level to vitamin D supplement use (**Fig. S4**).

High Time Outdoors Group
We first determined whether participants' blood sample used for vitamin D measurement was taken in the summer (March to September) or winter (October to February). To determine the relevant time spent outdoors, we used the variable "time spent outdoors in the summer" (field: 1050) for participants sampled in the summer, and for those sampled in winter we used the variable "time spent outdoors in the winter" (1060). Finally, participants that indicated spending 3hr or more outdoors in the season corresponding to season of blood draw were included.

Genetic scores
The 25OHD genetic scores were calculated in Plink2.0 based on the independent variants identified in the two genetic models (with and without interaction) from the White British population. A marginal score was calculated based on the beta coefficients in the marginal effects model, which was adjusted for CW-UVB but not for interaction. The variants included in this score were the independent COJO SNPs (N=105, median $\beta_{marginal}$= −0.013, IQR: −0.022,0.016; (1) marginal GRS = $\beta_{marginal}$*genotype). An interaction score was calculated based on the beta coefficients of the genetic main effect (β_G, median= −0.013, IQR: −0.033,0.022) and gene-CW-UVB interaction effect (β_{GxE}, median=$1.459\text{x}10^{-7}$, IQR: $-8.428\text{x}10^{-5}$, $5.388\text{x}10^{-5}$; (2) interaction score = β_G*genotype + β_{GxE}*genotype*average annual CW-D-UVB). The variants selected for this score were the independent variants identified in FUMA based on the joint p-value (N=238). Given that the interaction effect is dependent on genetic and environmental effects, β_{GxE} was additionally scaled by an average annual CW-UVB term for each individual: CW-UVB at the place of residence was averaged over a year up to the date of blood

sampling (median=283.3, IQR: 275.9, 301.7). The association of the genetic scores with measured 25OHD levels was evaluated in the European (excluding White British) group in the UKB (**Table S3**). The European group was identified based on the principal component analysis performed by the Pan-UK Biobank research group.[6] Imputed genetic data for this group was quality controlled for imputation quality, sex and genotype missingness, relatedness, and duplicates (N variants = 31,771,145; N participants = 24,235).

Sensitivity analysis
To evaluate whether the reported results were inflated with potential false positives or not adequately adjusted for confounding, we compared the significant independent results from the models reported in the main results (adjusted for CW-D-UVB, supplements, age, sex, and 10 PCs) to model 2 (further adjusted for 40 PCs) and model 3 (further adjusted for 40 PCs, birth location and assessment centre). Compared to model 2: there was no difference in the GxE test, 100% replicated; in the joint test 239 SNPs were identified compared to 238 in the original, 96% replicated (of which 221 were identical, 8 were in LD); and in the marginal test 103 SNPs compared to 105 in the original, 96% replicated (98 identical, 3 in LD). Compared to model 3: in the GxE test, 18 SNPs were identified compared to 20 in the original, 90% replicated (17 identical, 1 in LD), in the joint test 241 SNPs compared 238 in the original, 92% replicated (203 identical, 16 in LD); and in the marginal test, 104 SNPs were identified compared to 105 in the original, 92% replicated (88 identical, 9 in LD). The results from the marginal and GxE tests were largely unchanged while the number of significant SNPs increased in the joint test. Of the SNPs that did not replicate, many had not mapped to any gene in the original results (e.g. 8 of the joint SNPs) and key variants like those that mapped to *PTH*, *NPAS*, and *BMAL1* variants appeared as significant independent across models. Therefore, the original model were not likely to be inflated by false positives.

Sample size estimate for replication
We performed an analysis to determine the sample size required for replication of the variants identified in the discovery cohort, using the R package genpwr.[7] Sample size was estimated for the significant independent SNPs from the marginal model and from the joint model for each variant based on its effect size and minor allele frequency (from the discovery cohort). For the joint SNPs, β_G effect estimates were used, assuming a true model of additive effects and a 2 degrees of freedom joint test model. For the marginal SNPs, $\beta_{Marginal}$ effect estimates were used, assuming a true model of additive effects and an additive test model. For both models, the power threshold was set at 80% and the significance as a lenient significance threshold of 0.05 (genpwr.calc). We use $p < 0.05$ to estimate the minimum required sample size, noting that more stringent significance thresholds would require larger samples (e.g. the estimated sample size for a variant with MAF=0.1 and β=0.1 is approx. 4300 at $p < 0.05$ and 11,800 at $p < 0.00016$). The estimated sample sizes for a replication p-value of 0.05 ranged from N = 3,121 to N = 958,882 for the joint test and from 582 to 400,423 for the marginal (**Table S6**). The problem of replication is well-described in the literature wherein larger sample sizes are needed for replication than discovery to achieve enough power, given the constraint of testing for specific variants (see Liu et al.[8] for an in-depth discussion).

Replication cohorts
LURIC: The LURIC study consists of 3,316 patients of European ancestry hospitalized for coronary angiography between 1997 and 2000 at a tertiary care centre in Southwestern Germany. Clinical indications for angiography were chest pain or a positive non-invasive stress test suggestive of myocardial ischemia. To limit clinical heterogeneity, individuals suffering from acute illnesses other than acute coronary syndrome, chronic non-cardiac diseases and a history of malignancy within the past 5 years were excluded. The study was approved by the ethics committee at the "Ärztekammer Rheinland-Pfalz" and was conducted in accordance with the Declaration of Helsinki. Informed written consent was obtained from all participants. Genotyping in LURIC was done using the Affymetrix Genome-Wide Human SNP Array 6.0 and the TOPMed reference panel was used for genotype imputation. Variant liftover was performed by consensus between LiftOver and TableBrowser.[9] German postcode address data was converted to latitude and longitude coordinates for CW-D-UVB calculation.[10] Participants whose postcode could not be reliably converted were excluded (N = 15).

ORCADES: The Orkney Complex Disease Study (ORCADES) is a family-based study that seeks to identify genetic factors influencing cardiovascular and other disease risk in the isolated archipelago of the Orkney Isles in northern Scotland (McQuillan et al., 2008). 2078 participants aged 16-100 years were recruited between 2005 and 2011, most having three or four grandparents from Orkney, the remainder with two Orcadian grandparents. Fasting blood samples were collected and many health-related phenotypes and environmental exposures were measured in each individual. All participants gave written informed consent and the study was approved by Research Ethics Committees in Orkney, Aberdeen (North of Scotland REC), and South East Scotland REC, NHS Lothian (reference: 12/SS/0151). ORCADES is now part of Viking Genes (viking.ed.ac.uk), under South East Scotland REC, NHS Lothian (reference 19/SS/0104; IRAS 264868). The TOPMed reference panel was used for imputation and variant liftover was performed in the Ensembl assembly converter. Participants residing outside the UK were excluded (N = 4). Participants with 25OHD values below the lower detection limit (10 nmol/L) were also excluded (N = 108).

Supplementary Tables

Table S1: UK Biobank data fields

UKB variable (description)	Data-field
Age (age at recruitment, years)	21022
Sex	31
25 hydroxyvitamin D (nmol/L)	30890
Home location at assessment- east coordinate (1km resolution)	20074
Home location at assessment- north coordinate (1 km resolution)	20075
Time spend outdoors in summer	1050
Time spend outdoors in winter	1060
Body mass index (kg/m2)	21001
Vitamin and mineral supplements	6155
Mineral and other dietary supplements	6179

Table S2: Distribution of raw and standardised log-transformed 25OHD

Distribution of raw and standardised log-transformed 25OHD for all participants and in the White British group with vitamin D measurement (i.e. prior to exclusions on the basis of genetic QC or missing residential location data).

Participants	Summary statistic	25OHD	Stand. log transform. 25OHD
All participants (N=448,272)	Mean (SD)	48.6 (21.1)	$1.5x10^{-16}$ (1)
	Median	46.8	0.1
	IQR	32.4 – 62.4	-0.6 – 0.7
White British (N= 373,291)	Mean (SD)	49.84 (21.0)	0.0655 (0.969)
	Median	48.20	0.1995
	IQR	33.80 – 63.50	-0.5494 – 0.7813

Table S3. Association of 25OHD with potential covariates
Association of 25OHD with potential covariates in all UKB participants with available 25OHD data (N=448,272) overall and in the White British group (prior to excluding participants for genetic QC or missing address data). Beta values are shown from a simple unadjusted linear regression, 25OHD ~ covariate and p-values from a two-sided test.

Participants	Covariate	Prop or Mean (SD)	25OHD - Beta (SE)	p-value	Stand. log transform. 25OHD - Beta (SE)	p-value
All participants (N=448,272)	Age	56.5 (8.12)	0.2496 (-0.0038)	0	0.0134 (-0.0001)	0
	Sex (M)	0.46	-0.1516 (-0.0632)	0.0165	-0.0105 (-0.0029)	0.0004
	UVB	2.29 (2.09)	1.8618 (-0.0148)	0	0.0935 (-0.0007)	0
	CW-D-UVB	98.89 (71.8)	0.1036 (-0.0004)**	0	0.005 (0)	0
	Fish oil supplement	0.31	9.4625 (-0.0665)	0	0.4732 (-0.0031)	0
	Vitamin supplement	0.24	8.3143 (-0.0723)	0	0.4049 (-0.0034)	0
White British (N=373,291)	Age	56.8 (8.04)	0.1898 (-0.0042)	0	0.0099 (-0.0001)	0
	Sex (M)	0.47	0.085 (-0.0688)	0.2168	0.0036 (-0.0031)	0.2521
	UVB	2.26 (2.07)	2.0118 (-0.0163)	0	0.0996 (-0.0007)	0
	CW-D-UVB	98.29 (71.5)	0.1099 (-0.0004)	0	0.0053 (0)	0
	Fish oil supplement	0.32	9.354 (-0.0722)	0	0.4576 (-0.0033)	0
	Vitamin supplement	0.24	8.701 (-0.0795)	0	0.4142 (-0.0036)	0

Table S4: Association of genetic scores with 25OHD
Association of each genetic score with 25OHD in the **European** population, based on an age and sex adjusted linear model, two-sided p-values are shown.

	Coefficient (SE)	p-value	Adjusted R^2
**Marginal model (25OHD ~ age + sex + marginal score)**			
age	0.195 (0.017)	$7.82x10^{-32}$	
sex	-0.318 (0.274)	0.247	
marginal score	14.319 (0.485)	$2.79x10^{-188}$	0.04363
**Interaction model (25OHD ~ age + sex + interaction score)**			
age	0.197 (0.017)	$1.63x10^{-31}$	
sex	-0.278 (0.278)	0.317	
interaction score	5.210 (0.214)	$9.91x10^{-130}$	0.03227

Supplementary sheet tables
The supplementary sheet includes the following tables:
Table S5: Independent significant variants based on the results from the White British group in GEM, including effect estimates and p-values.
Table S6: Results of the genpwr and replication analyses.
Table S7: LDSC SNP heritability estimates.
Table S8: FUMA gene annotation based on the joint test results.
Table S9: FUMA lead SNP annotation based on the joint test results.
Table S10: MAGMA gene-set annotation based on the joint test results.
Table S11: MAGMA tissue annotation based on the joint test results.
Table S12: FUMA gene-set annotation based on the interaction test results
Table S13: FUMA (ANNOVAR) functional consequences of joint SNPs
Table S14: LDSC genetic correlation of 25OHD with other traits

Supplementary Figures

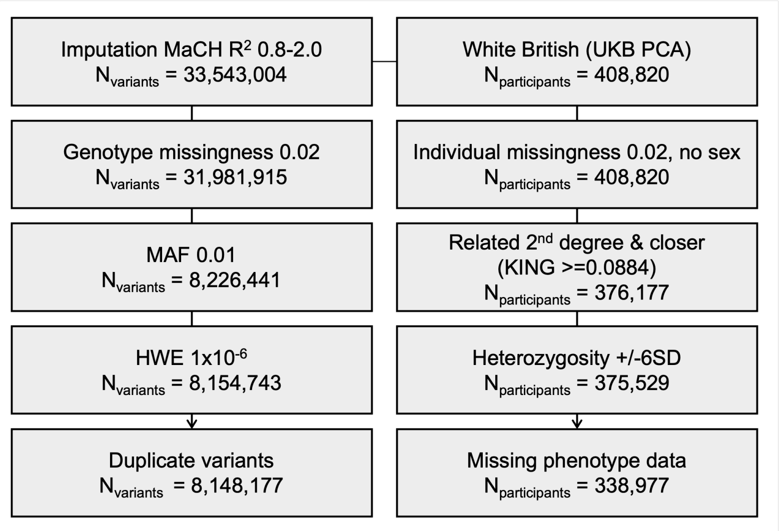

Figure S1: Quality control
Quality control workflow performed in Plink2, with the number of variants or participants remaining following each step.

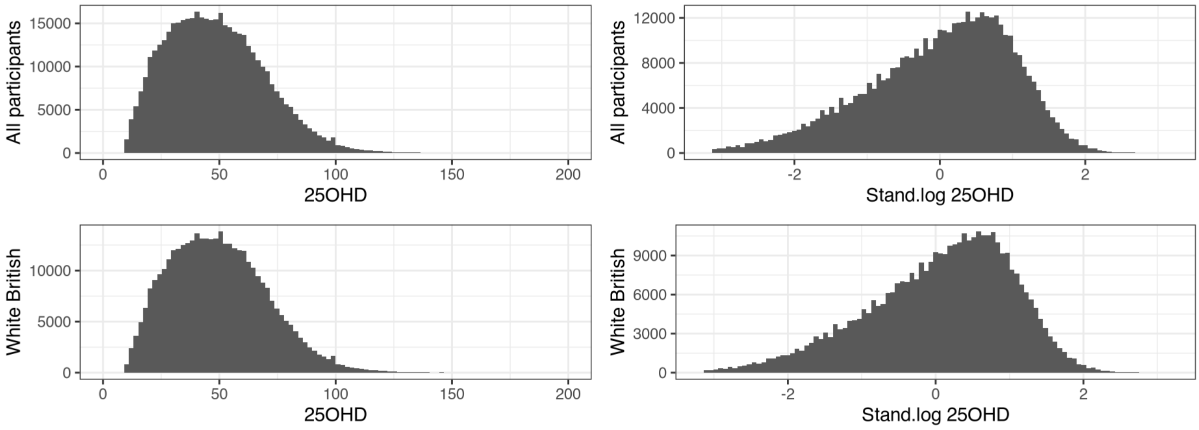

Figure S2: Distribution of 25OHD
Distribution of 25OHD in UK Biobank overall and within the White British group before (**left**) and after log-transformation and standardisation (**right**).

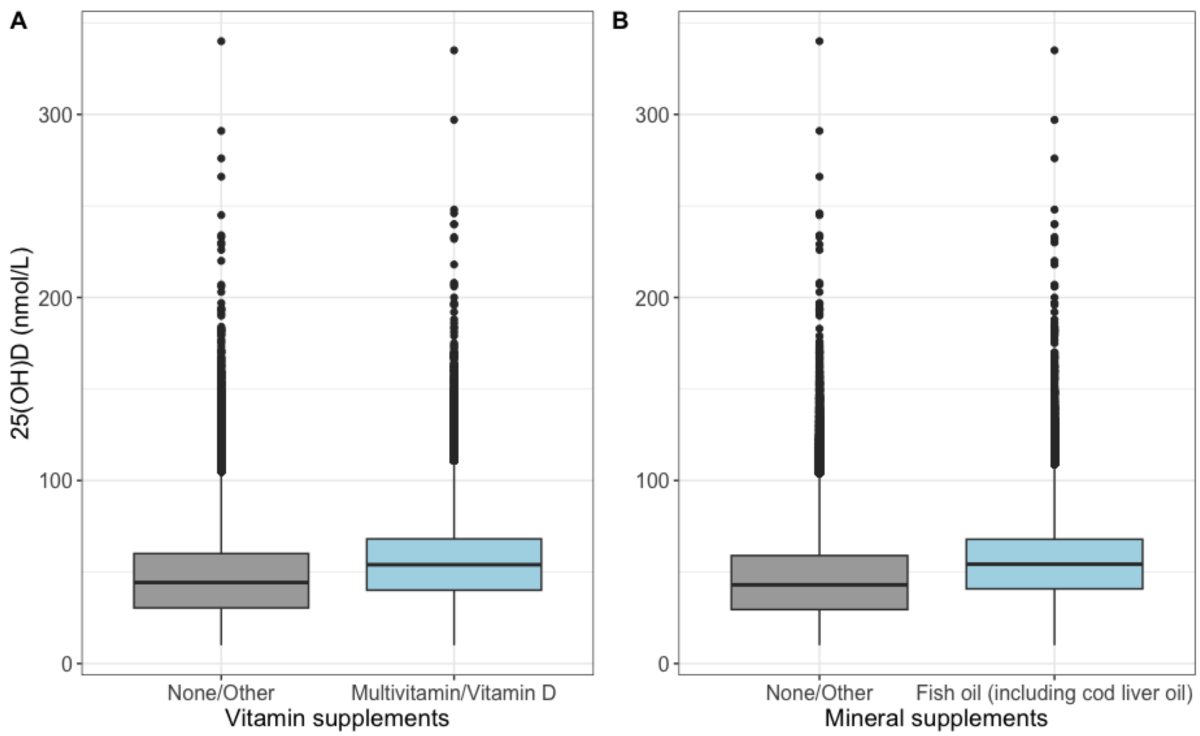

Figure S3: Supplement and fish oil intake

The difference in 25OHD concentration in participants **(A)** who do/do not take multivitamins and/or vitamin D supplements and **(B)** who do/do not take or fish oils. See Brennan et. al.[11] for an in depth evaluation of vitamin D modifiers in the UKB.

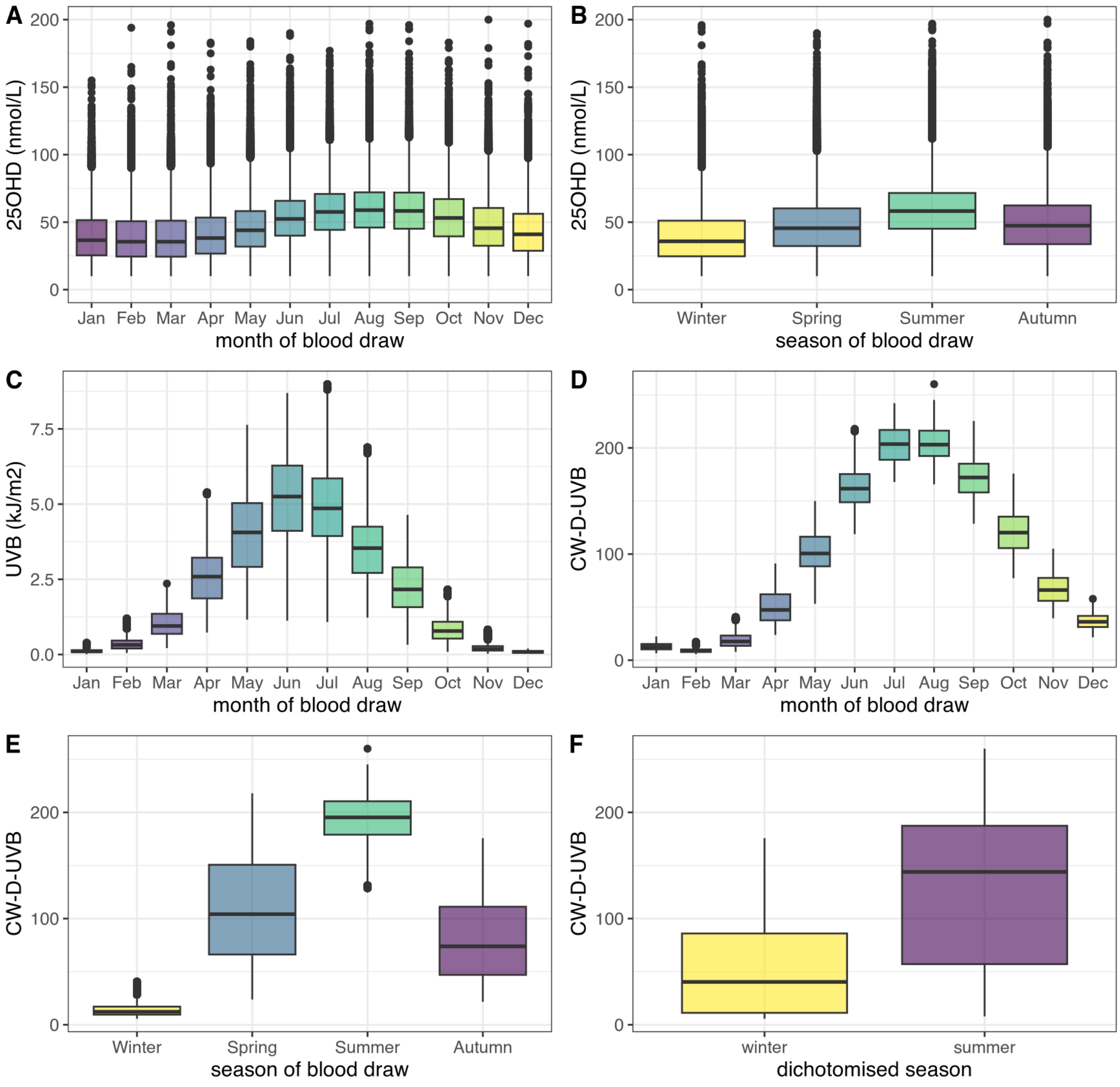

Figure S4: Distribution of vitamin D and UVB

The distribution of 25OHD (nmol/L) in UK Biobank by **(A)** month of blood draw and **(B)** season of blood draw. The y-axis is limited to 200 nmol/L for readability in A and B (approx. 0.006% of participants have 25OHD > 200 nmol/L). The distribution of **(C)** daily D-UVB (kJ/m^2) and **(D)** cumulative weighted D-UVB (CW-D-UVB) by month of blood draw. The distribution of CW-D-UVB by season of blood draw **(E)** and when season is dichotomised to winter (October-February) and summer (March-September) **(F)**.

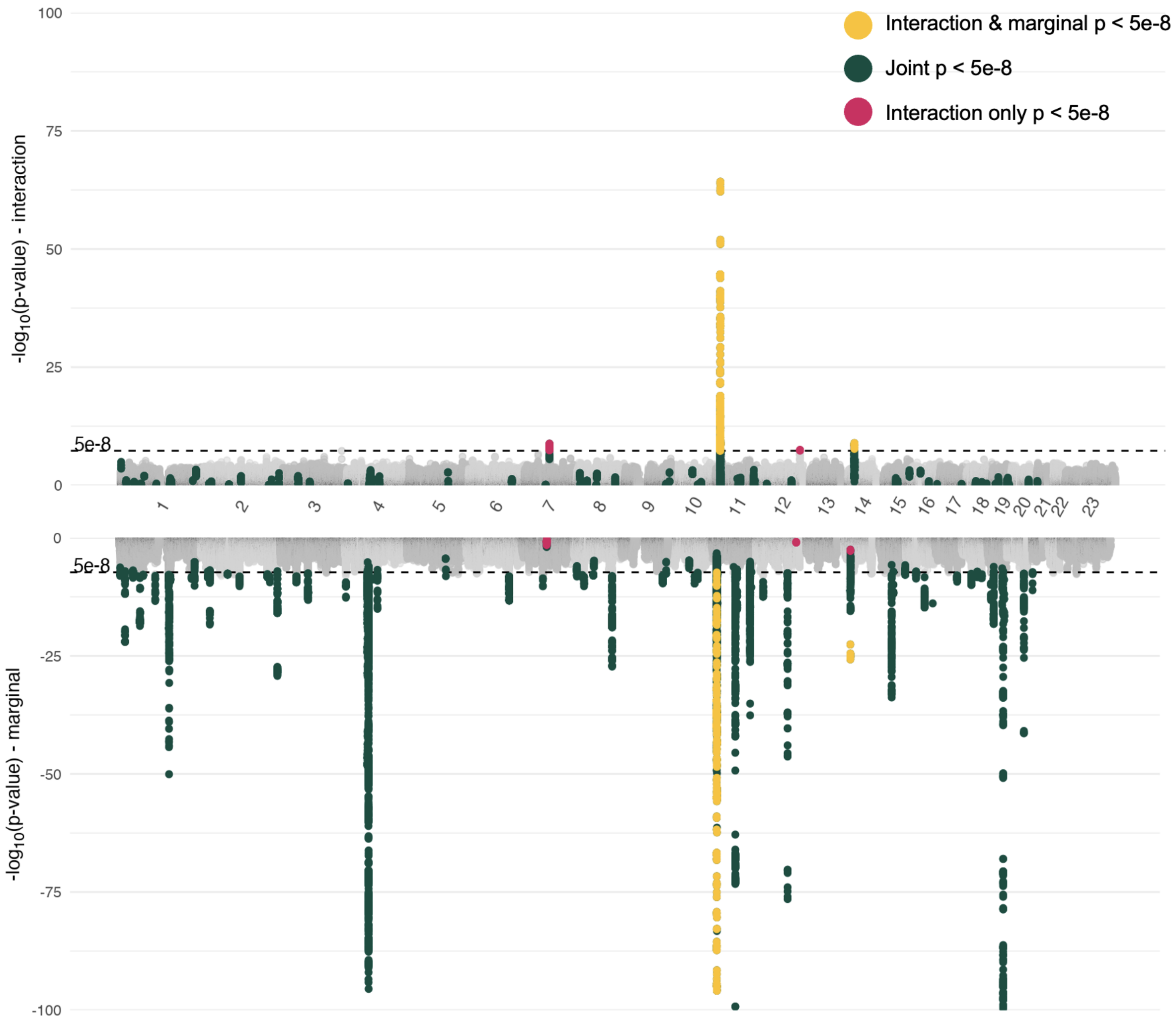

Figure S5: Mirrored Manhattan plot of genome-wide GxE and marginal tests
Mirrored Manhattan plot of the genome-wide gene-environment interaction study of standardised, log-transformed 25OHD in the UK Biobank. The **top** Manhattan plot shows the p-values from the interaction test while the **bottom** plot shows the p-values from the marginal test (main effects only, no interaction). The p-values are shown on the -log10 scale and the dashed line shows the significance cutoff line of $P < 5 \times 10^{-8}$ from the GEM analysis interaction test. Chromosome numbers are shown on the x-axis, where 23 represents the X-chromosome. The dots highlighted in yellow represent the variants identified as significant in the interaction and marginal tests, pink variants were significant only on the interaction test, and green variants were significant on the joint test. The vertical axis is limited to 100 for readability.

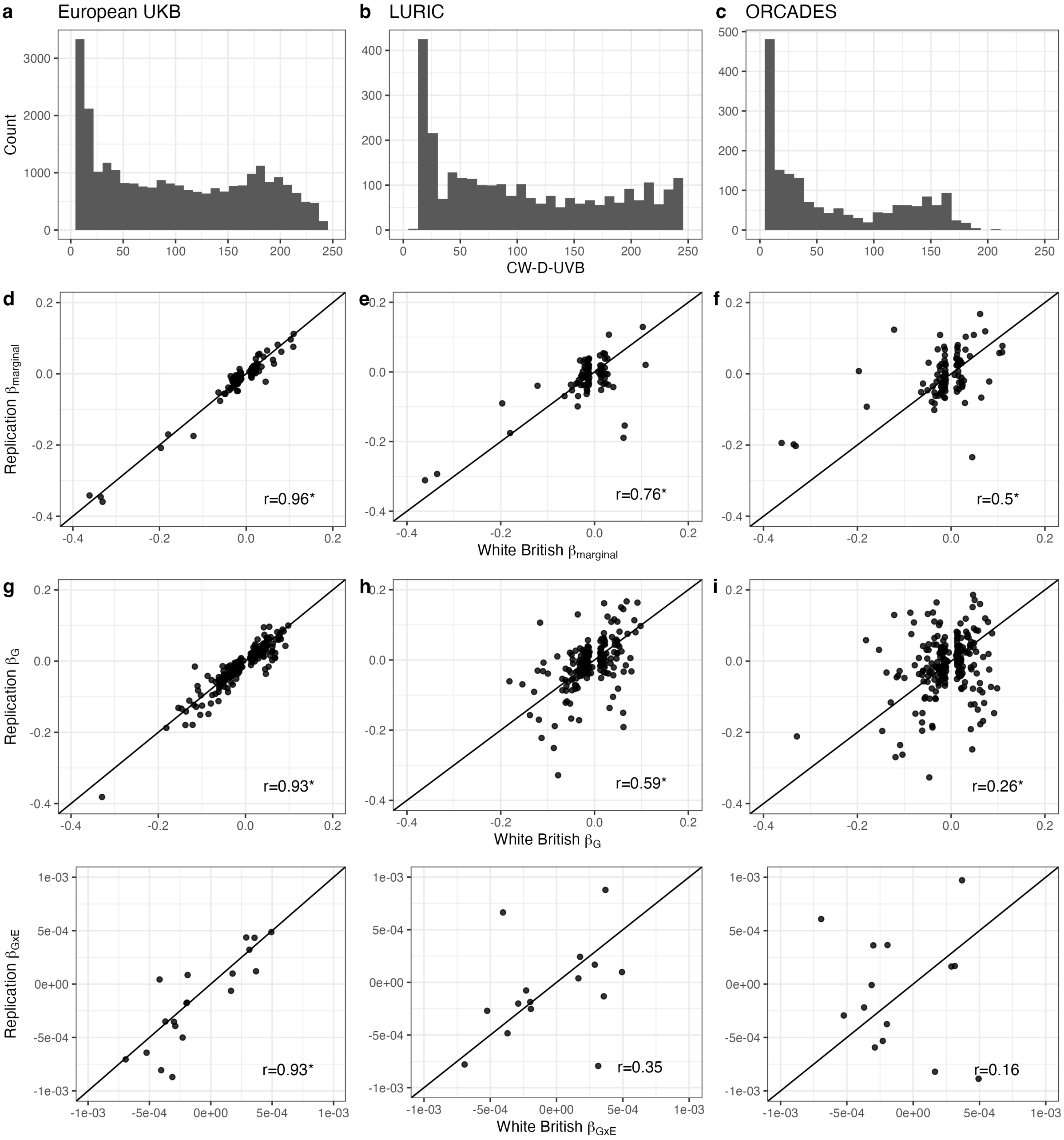

Figure S6: Correlation of effect estimates in discovery & replication cohorts

The distribution of CW-D-UVB is shown within each of the three replication cohorts (1st column European, N=24,235; 2nd column LURIC, N=2,909; and 3rd column ORCADES, N=1,875). Additionally, comparisons of the effect estimates from the main analysis (White British, N=338,977) and the three replication cohorts are shown. Significant correlation coefficients (r) are marked with an asterisk (*).

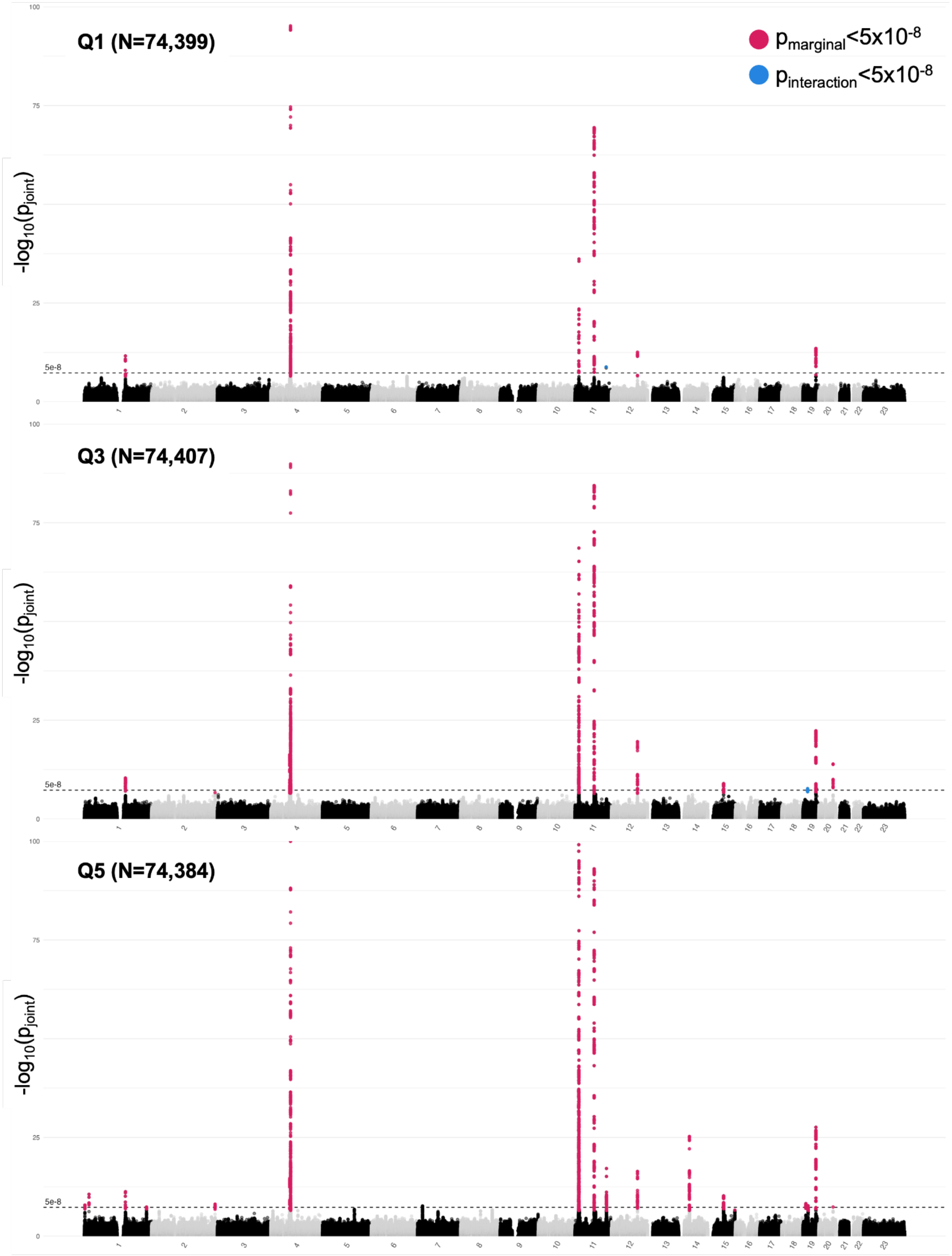

Figure S7: Manhattan plots by CW-D-UVB quintile

Manhattan plot based on the joint test p-values of standardised log-transformed 25OHD in the first, third, and fifth quintiles of CW-D-UVB among White British participants. The total significant variants (p-value < $5x10^{-8}$) for each test within each quintile were as follows. In Q1: 1 interaction (chr11:rs11215670), 1,420 marginal, 1,364 joint; in Q2: 1,893 marginal, 1,812 joint, and no interaction variants; in Q3: 7 interaction, 3,017 marginal, 2,771 joint; in Q4: 3,528 marginal, 3,037 joint and no interaction variants; and in Q5: 4,117 marginal, 3,903 joint, and no interaction variants.

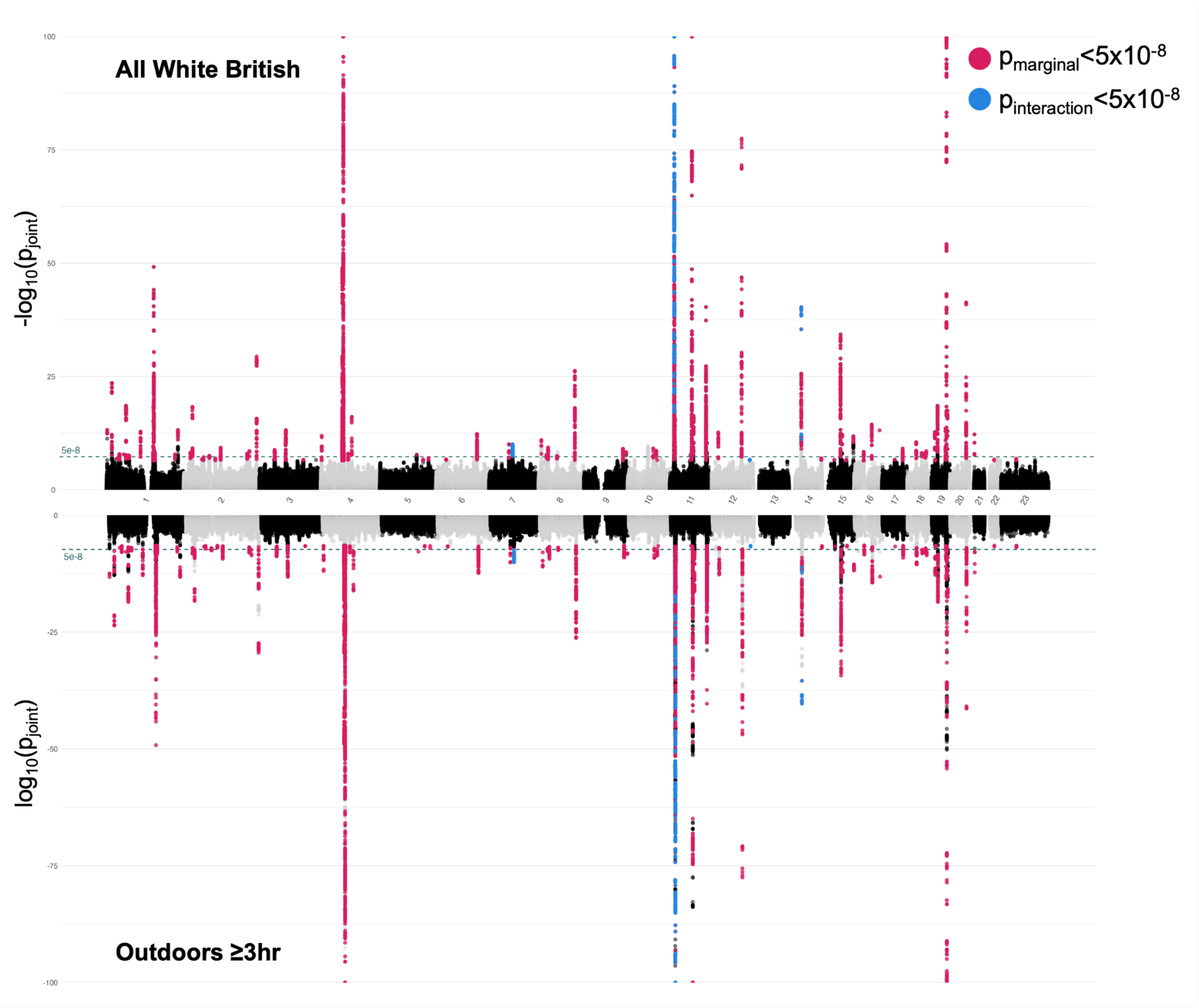

Figure S8: Manhattan plot of high time outdoors subgroup.
Mirrored genome-wide gene-environment interaction p-values of standardised log-transformed 25OHD in all White British participants (N=375,435, **top**) compared to participants who indicated that they spent 3hr or more outdoors (N=172,273, **bottom**). The y-axis shows the joint p-value, highlighted variants indicate genome-wide significant $p_{interaction}$ or $p_{marginal}$ ($p<5x10^{-8}$). In the outdoor group, 6,371 variants were significant on the marginal test, 524 interaction, and 6,569 joint (compared to 953 interaction, 11,511 marginal, and 11,717 joint variants in the total White British sample).

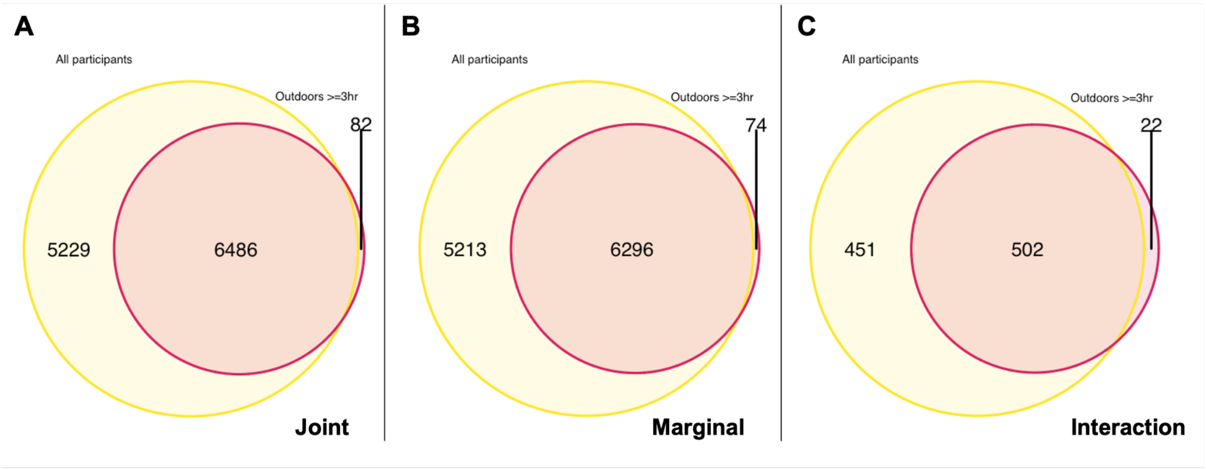

Figure S9: Venn diagram of overlap with outdoors subgroup.
Overlap of significant joint (A), marginal (B) and interaction (C) variants in the analysis of the entire cohort and the outdoors groups.

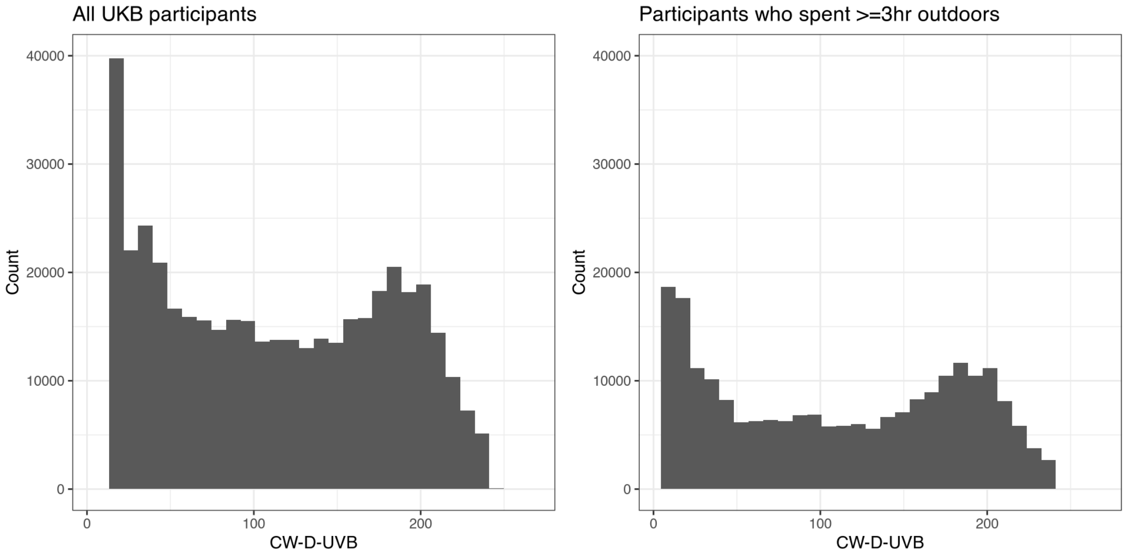

Figure S10: Distribution of CW-D-UVB in outdoors subgroup
Distribution of CW-D-UVB across all participants (median=87.806, IQR: 27.249, 165.239), and in participants who spend 3hr or more outdoors in the season of sampling (median=111.671, IQR: 37.713, 178.203).

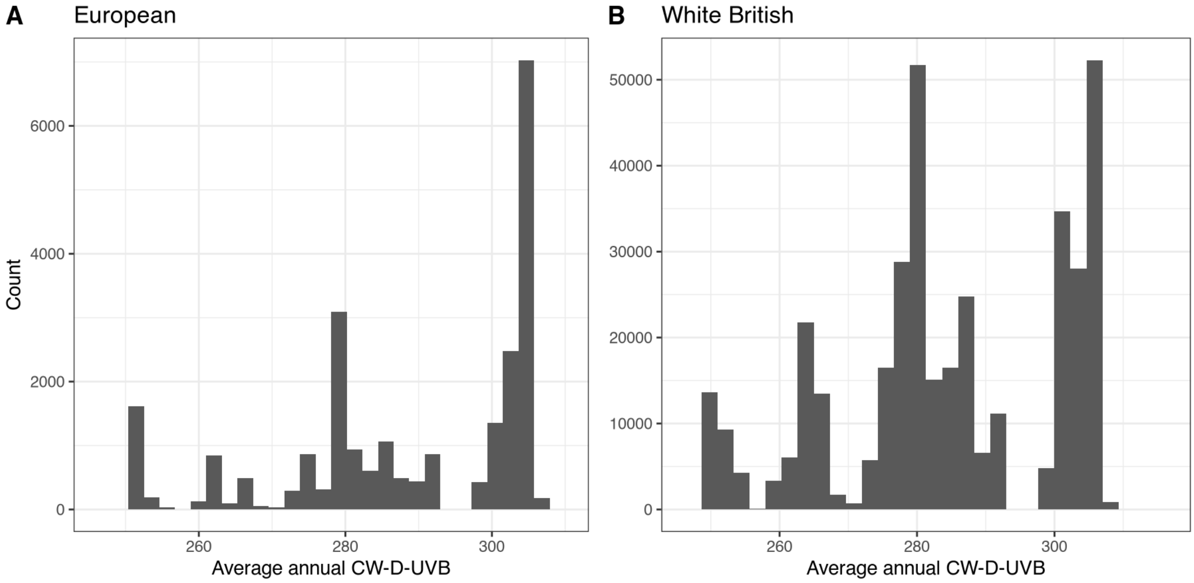

Figure S11: Distribution of CW-D-UVB in the European cohort
Average annual CW-D-UVB at the participant's place of residence in the European (A) and White British (B) participants, averaged across a year prior to the date of blood sample.

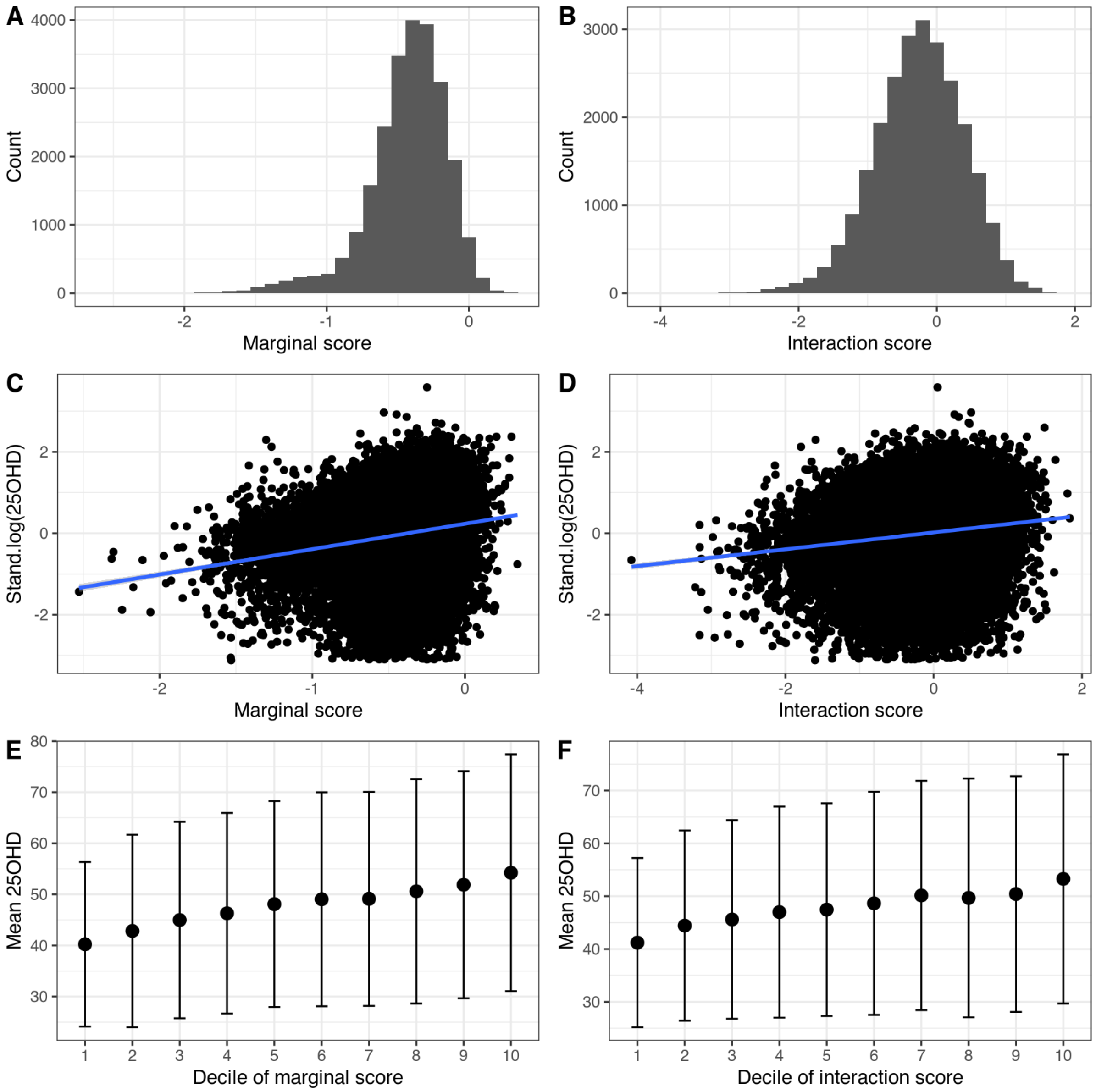

Figure S12: Distribution of genetic scores and association with 25OHD
Distribution of the **(A)** marginal and **(B)** interaction genetic risk scores in the European cohort (N=24,234). Association of **(C)** the marginal score and **(D)** the interaction score with standardised log-transformed 25OHD. Change in mean 25OHD by **(E)** decile of marginal score and **(F)** decile of interaction score (N=23,768 after removing missing 25OHD).

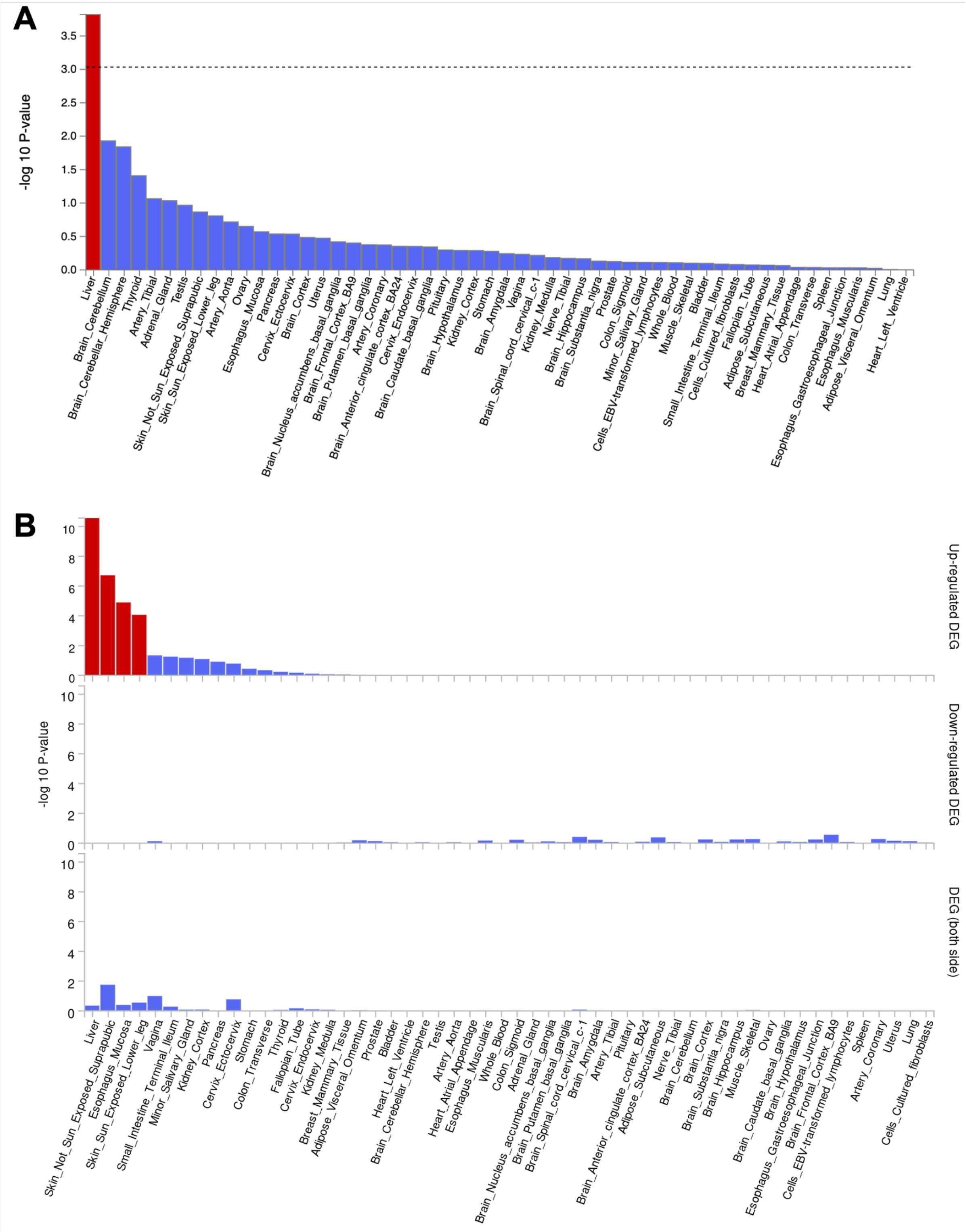

Figure S13: MAGMA tissue expression analysis

MAGMA tissue expression analysis in FUMA based (**A**) on the full distribution of SNP p-values in the 25OHD joint test summary statistics (SNP2GENE) and (**B**) on the prioritised genes based on differentially expressed gene sets (GENE2FUNC). $P_{Bonferroni} < 0.05$ are highlighted in red.

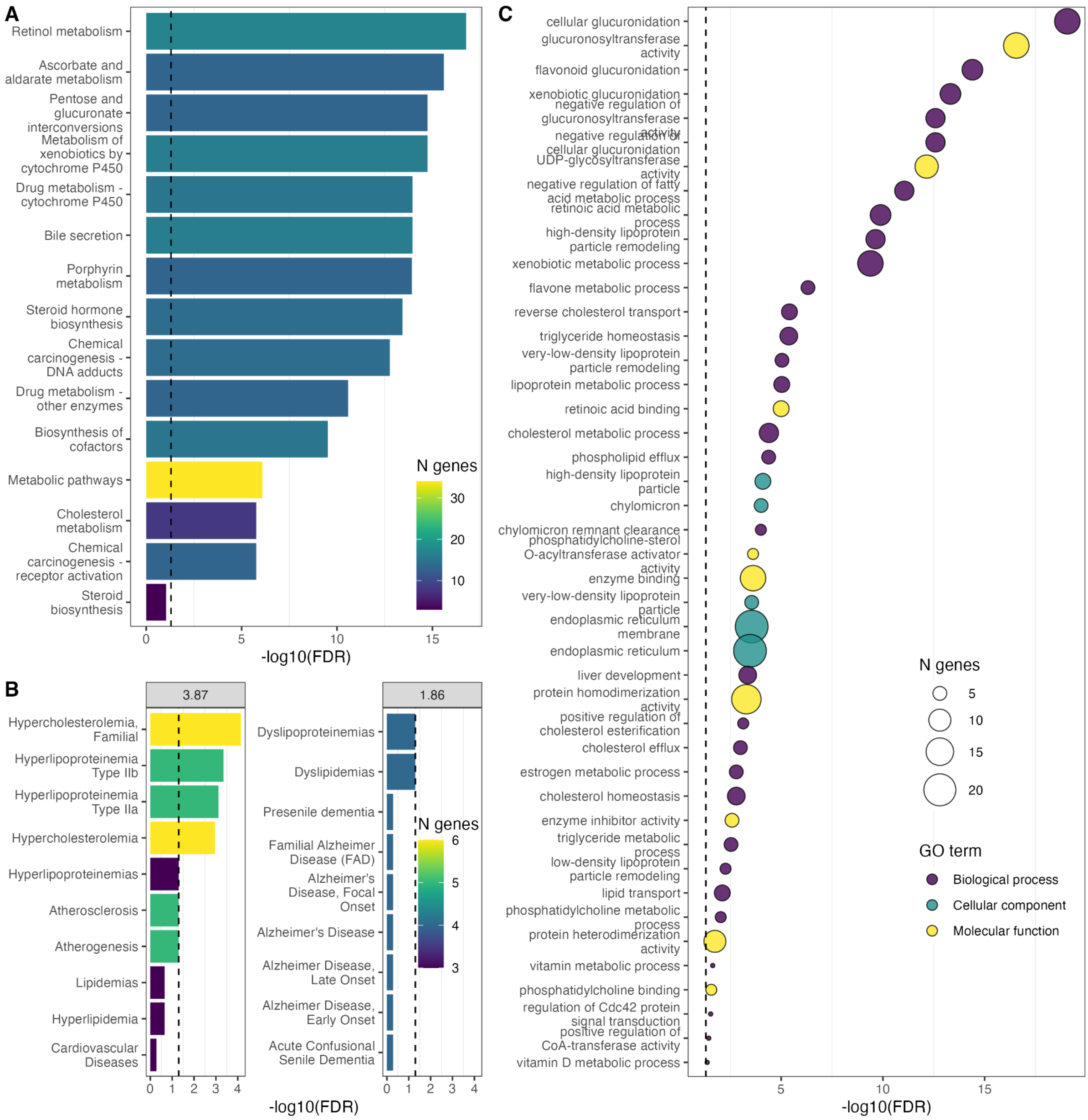

Figure S14: DAVID functional annotation

Functional annotation in DAVID of the 148 genes mapped to the marginal, interaction, and/or joint significant independent variants (EASE threshold 0.05). (**A**) Enrichment of KEGG pathways. Dashed line represents 0.05 FDR and the count scale shows the number of genes in each pathway. **(B)** Enrichment of DISGENET disease terms. Disease terms are clustered to minimise redundant annotations, only the top two clusters are shown, the enrichment score is at the top. Dashed line represents 0.05 FDR and the count scale shows the number of genes in each pathway. (**C**) Enrichment of GO terms corresponding to biological processes, cellular components, and molecular functions. Colour corresponds to the GO term type (BP: biological process, CC: cellular component, MF: molecular function) and size to the number of genes linked to that GO term. FDR is on the y-axis, only GO terms with FDR < 0.05 are shown.

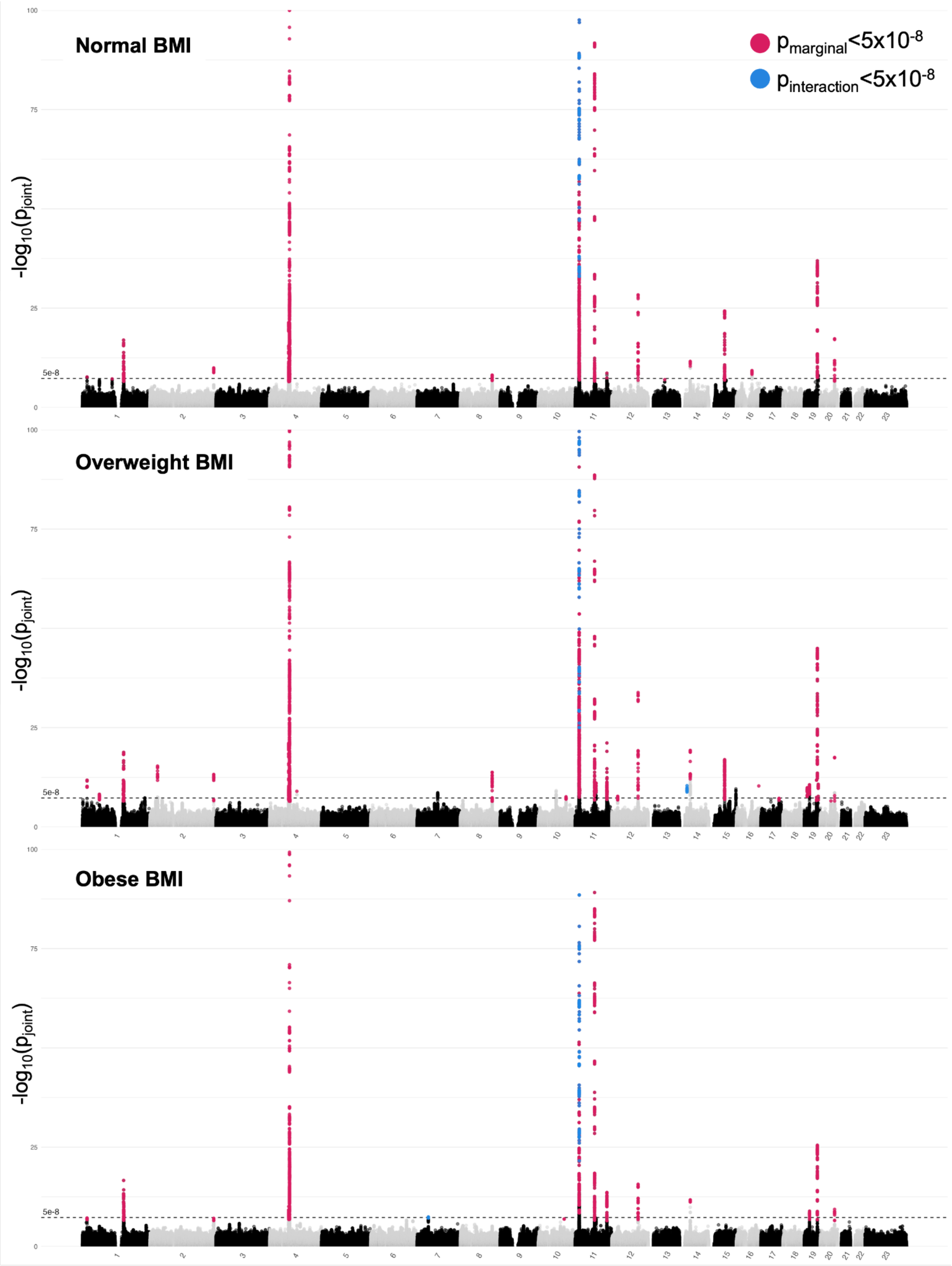

Figure S15: Manhattan plots by BMI category

Manhattan plot of the joint test p-values of standardised log-transformed 25OHD, stratified by BMI categories: normal 18.5-24.99 kg/m^2 (N=120,925), overweight 25-29.99 kg/m^2 (N=159,671), and obese ≥30 kg/m^2 (N=91,997). The total significant variants (p-value < 5x10^{-8}) for each test within each stratum were as follows. In normal BMI: 4,098 marginal, 256 interaction, 4,287 joint; in the overweight group: 4,937 marginal, 291 interaction, 5,412 joint; and in the obese group: 2,548 marginal, 191 interaction, and 2,533 joint.

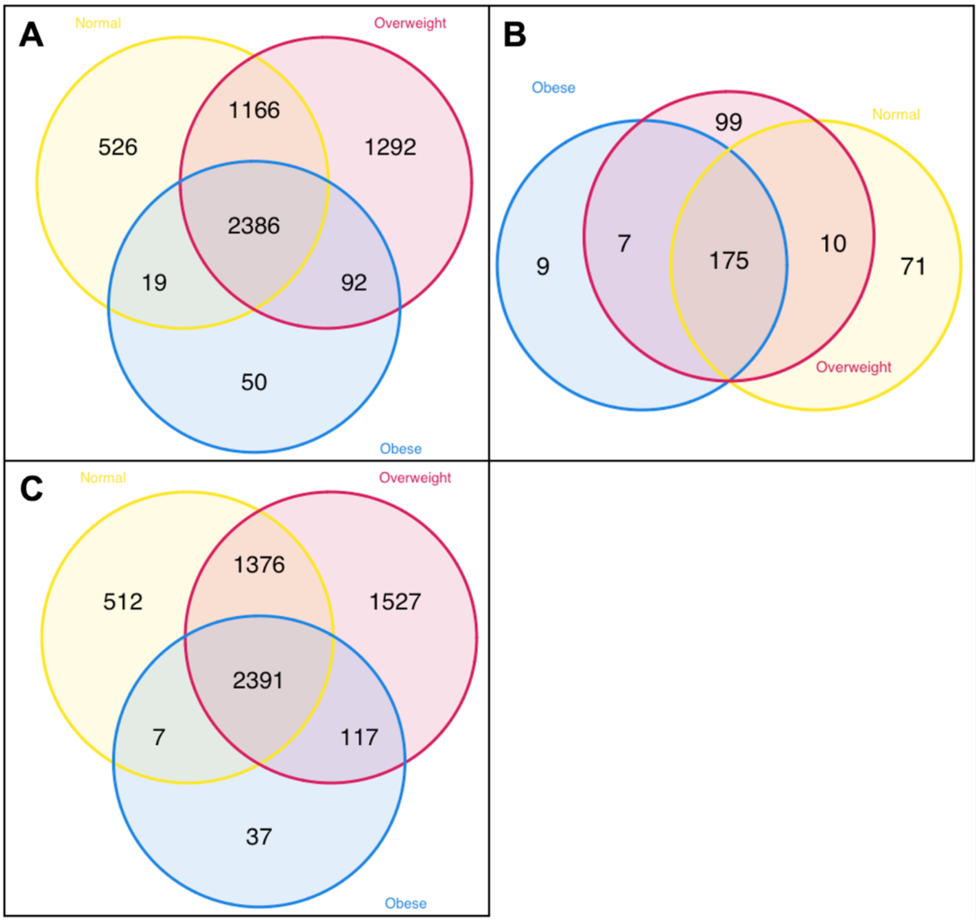

Figure S16: Venn diagram of overlap across BMI categories

Overlap of genome-wide significant marginal (A), and interaction (B), and joint (C) variants in the BMI categories, normal (N=120,925), overweight (N=159,671), and obese (N=91,997).

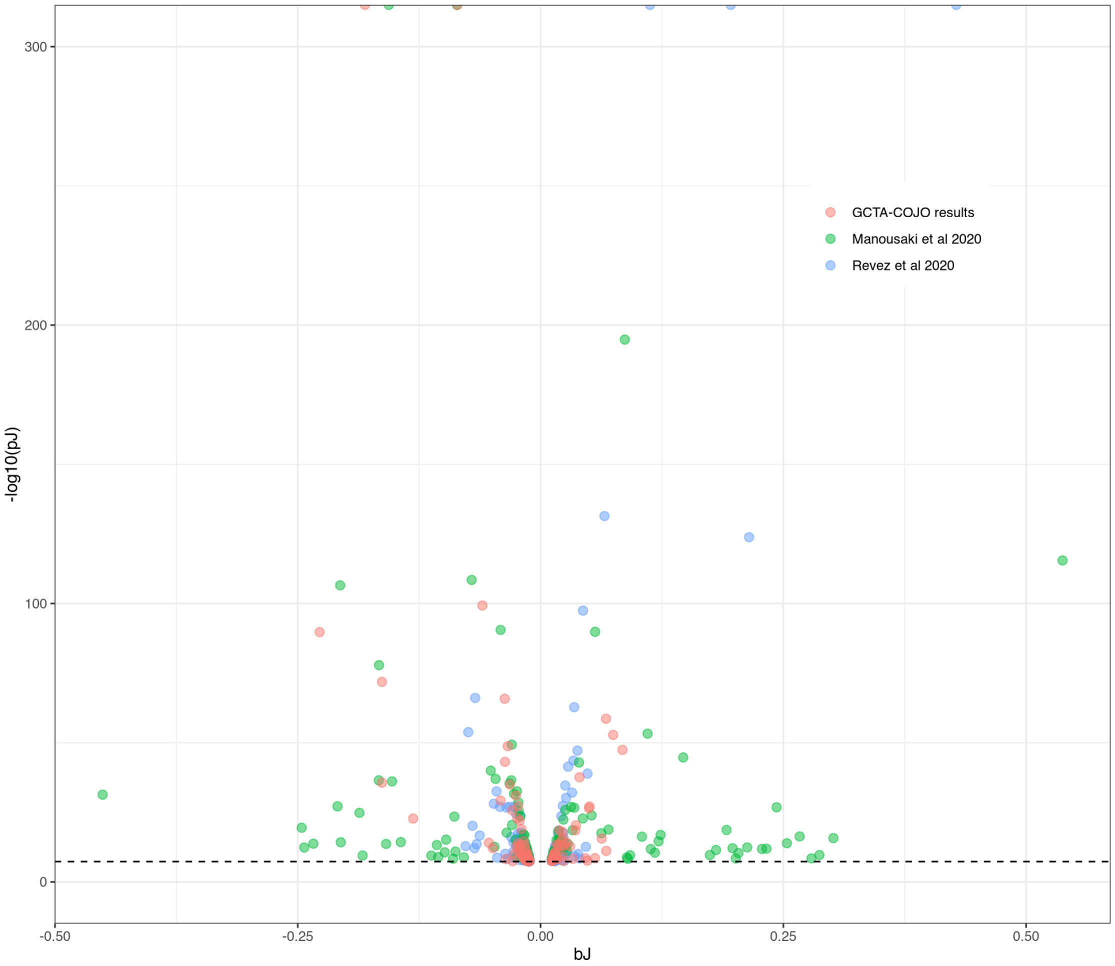

Figure S17: Effect estimate and p-value comparison with recent GWAS
Comparison of our results from the White British population with the previous vitamin D GWAS studies interrogating the same phenotype in UK Biobank. The results shown are from the GCTA-COJO conditional analysis. The y-axis shows -$\log_{10}$(p-values) and the x-axis shows the joint beta effect estimates from the COJO output. The dashed line represents the p-value cutoff $5x10^{-8}$. The spread of our results (pink) is similar to those from the Revez et. al., 2020 (blue) study with a narrower spread of effect estimates. Comparatively, the variants from the Manousaki et. al., 2020 (green) have larger effect estimates but less significant p-values.

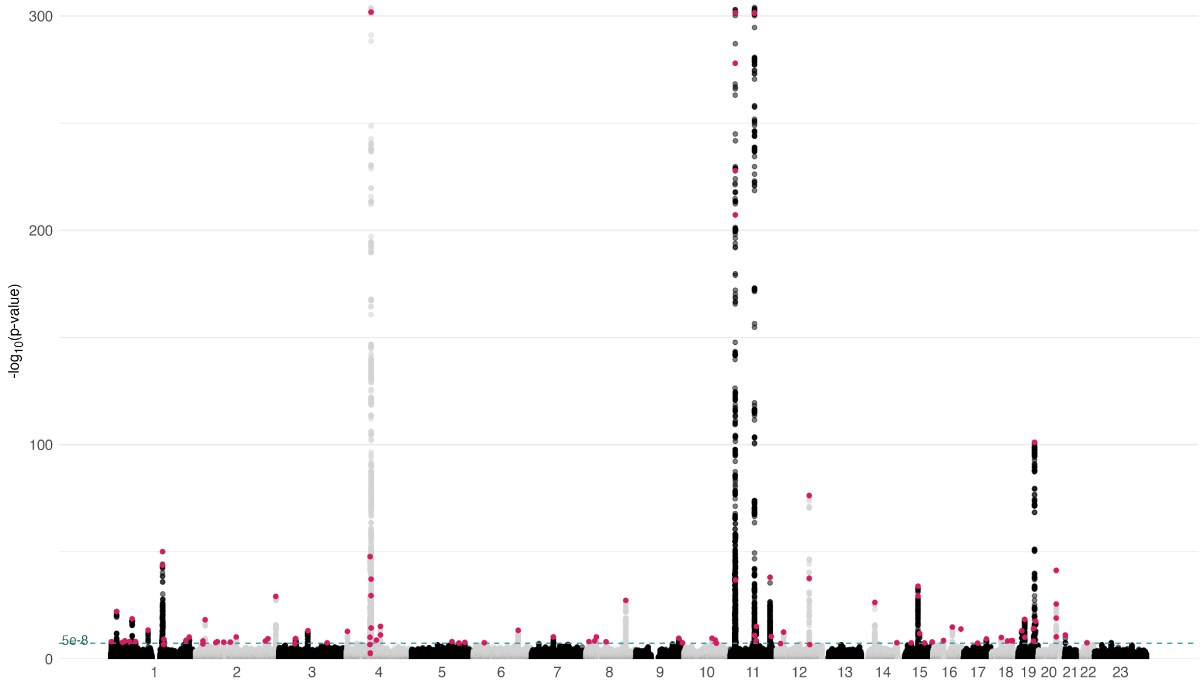

Figure S18: Manhattan plot of Plink2 GWAS results

Manhattan plot of the genome-wide association study of standardised, log-transformed 25OHD in the UK Biobank using Plink2. The p-values are shown on the -log10 scale and the dashed line shows the significance cutoff line of $P < 5 \times 10^{-8}$ from the Plink2 association test. Independent significant variants selected with GCTA-COJO are highlighted in pink. Chromosome numbers are shown on the x-axis, where 23 represents the X-chromosome. The results are largely comparable to the GEM results reported in the main text. The results from both tools are largely comparable. Briefly, the Plink2 GWAS, adjusted for the same covariates including CW-D-UVB, identified a total of 11,482 genome-wide significant variants associated with 25OHD at p-value $< 5 \times 10^{-8}$. We then similarly applied COJO-GCTA[12] to the GWAS summary statistics and identified 105 significant independent SNPs, at pJ $< 5 \times 10^{-8}$, 15 of these variants were low frequency variants, MAF < 0.05 (GEM results: 11,511 variants with $p_{marginal} < 5 \times 10^{-8}$; 105 significant independent variants identified by COJO, see 'Results'). All but two variants from the marginal GEM results were replicated, one of which was in high LD with a GEM variant.

References

1. Chang, C.C. *et al.* Second-generation PLINK: rising to the challenge of larger and richer datasets. *Gigascience* **4**, 7 (2015).
2. Bouillon, R. *et al.* Action spectrum for the production of previtamin D3 in human skin. *UDC* **612**, 481-506 (2006).
3. Zempila, M.M. *et al.* TEMIS UV product validation using NILU-UV ground-based measurements in Thessaloniki, Greece. *Atmos. Chem. Phys.* **17**, 7157-7174 (2017).
4. Kelly, D. *et al.* The contributions of adjusted ambient ultraviolet B radiation at place of residence and other determinants to serum 25-hydroxyvitamin D concentrations. *Br J Dermatol* **174**, 1068-78 (2016).
5. Jones, G. Pharmacokinetics of vitamin D toxicity. *Am J Clin Nutr* **88**, 582S-586S (2008).
6. Konrad J. Karczewski *et al.* Pan-UK Biobank GWAS improves discovery, analysis of genetic architecture, and resolution into ancestry-enriched effects. *medRxiv* (2024).
7. Moore, C.M., Jacobson, S.A. & Fingerlin, T.E. Power and Sample Size Calculations for Genetic Association Studies in the Presence of Genetic Model Misspecification. *Hum Hered* **84**, 256-271 (2019).

8. Liu, Y.J., Papasian, C.J., Liu, J.F., Hamilton, J. & Deng, H.W. Is replication the gold standard for validating genome-wide association findings? *PLoS One* **3**, e4037 (2008).
9. Hinrichs, A.S. *et al.* The UCSC Genome Browser Database: update 2006. *Nucleic Acids Res* **34**, D590-8 (2006).
10. Konrad, M. Geocoordinates for German postal codes ("PLZ"). https://github.com/WZBSocialScienceCenter/plz_geocoord (2019).
11. Brennan, M.M., van Geffen, J., van Weele, M., Zgaga, L. & Shraim, R. Ambient ultraviolet-B radiation, supplements and other factors interact to impact vitamin D status differently depending on ethnicity: A cross-sectional study. *Clin Nutr* **43**, 1308-1317 (2024).
12. Yang, J. *et al.* Conditional and joint multiple-SNP analysis of GWAS summary statistics identifies additional variants influencing complex traits. *Nat Genet* **44**, 369-75, S1-3 (2012).
