# Supplementary material for: Genome-wide gene-environment interaction study uncovers 162 vitamin D status variants using a precise ambient UVB measure
Source: Nat Commun. 2025 Nov 28;16:10774. doi: 10.1038/s41467-025-65820-x (PMC12663108; doi:10.1038/s41467-025-65820-x)
Supplement: Supplementary file 3 — Reporting Summary [file 41467_2025_65820_MOESM3_ESM.pdf]

nature portfolio

Corresponding author(s): Lina Zgaga

Last updated by author(s): 13/8/2025

Reporting Summary

Nature Portfolio wishes to improve the reproducibility of the work that we publish. This form provides structure for consistency and transparency in reporting. For further information on Nature Portfolio policies, see our Editorial Policies and the Editorial Policy Checklist.

Statistics

For all statistical analyses, confirm that the following items are present in the figure legend, table legend, main text, or Methods section.

n/a	Confirmed	
☐	☒	The exact sample size (*n*) for each experimental group/condition, given as a discrete number and unit of measurement
☐	☒	A statement on whether measurements were taken from distinct samples or whether the same sample was measured repeatedly
☐	☒	The statistical test(s) used AND whether they are one- or two-sided<br>*Only common tests should be described solely by name; describe more complex techniques in the Methods section.*
☐	☒	A description of all covariates tested
☐	☒	A description of any assumptions or corrections, such as tests of normality and adjustment for multiple comparisons
☐	☒	A full description of the statistical parameters including central tendency (e.g. means) or other basic estimates (e.g. regression coefficient) AND variation (e.g. standard deviation) or associated estimates of uncertainty (e.g. confidence intervals)
☐	☒	For null hypothesis testing, the test statistic (e.g. *F*, *t*, *r*) with confidence intervals, effect sizes, degrees of freedom and *P* value noted<br>*Give P values as exact values whenever suitable.*
☒	☐	For Bayesian analysis, information on the choice of priors and Markov chain Monte Carlo settings
☒	☐	For hierarchical and complex designs, identification of the appropriate level for tests and full reporting of outcomes
☐	☒	Estimates of effect sizes (e.g. Cohen's *d*, Pearson's *r*), indicating how they were calculated

Our web collection on statistics for biologists contains articles on many of the points above.

Software and code

Policy information about availability of computer code

Data collection	UK Biobank data was obtained by direct application to the UK Biobank (Application 73479). UVB data was obtained from the TEMIS database. GWAS summary statistics for other traits are publicly available (links/accession codes provided below).
Data analysis	GEM 1.5 was used for the genetic analysis and is available through the software author's GitHub page (https://github.com/large-scale-gxe-methods/GEM). Other analyses were performed using R version 4.3.2, Plink1.9, Plink2, GCTA1.94 (COJO), LDSC, and FUMA online platform. The R code for generating UV dose is available on https://github.com/rshraim/UVdose.

For manuscripts utilizing custom algorithms or software that are central to the research but not yet described in published literature, software must be made available to editors and reviewers. We strongly encourage code deposition in a community repository (e.g. GitHub). See the Nature Portfolio guidelines for submitting code & software for further information.

Data

Policy information about availability of data

All manuscripts must include a data availability statement. This statement should provide the following information, where applicable:

- Accession codes, unique identifiers, or web links for publicly available datasets
- A description of any restrictions on data availability
- For clinical datasets or third party data, please ensure that the statement adheres to our policy

Summary statistics for the marginal, interaction, joint, and stratified (BMI, CW-D-UVB quintiles, time outdoors) analyses are available from the GWAS Catalog

(GCST90652548:GCST90652559). The CW-D-UVB dose is available through the UK Biobank returned results. This research has been conducted using the UK Biobank Resource under Application Number 73479 and participant data is available by application to the UK Biobank. UV data is available at www.temis.nl/uvradiation. GWAS summary statistics for genetic correlation can be downloaded from the GWAS Catalog (study accession) https://www.ebi.ac.uk/gwas/ for multiple sclerosis (GCST005531), melanoma skin cancer (GCST90011809), circadian rhythm (GCST003837), asthma (GCST010042), osteoporosis (GCST90018887), Parkinson's disease (GCST009325), colorectal cancer (CRC; GCST9012950, Alzheimer disease (GCST90027158), intelligence (GCST004364), breast cancer (GCST007236), BMI (GCST90179150), DBP (GCST90000059), forearm fracture (GCST90281273), and SBP (GCST90000062); from the Psychiatric Genetics Consortium https://pgc.unc.edu/for-researchers/download-results/ for autism spectrum disorder (asd2019), schizophrenia (scz2022), bipolar disorder (bip2021), major depression disorder (mdd2018), and anxiety (anx2016); from https://plaza.umin.ac.jp/yokada/datasource/software.htm for rheumatoid arthritis; from https://cncr.nl/research/summary_statistics/ for sensitivity to environmental stress and adversity (SESA); from the Program in Complex Trait Genomics https://cnsgenomics.com/content/data for type 2 diabetes; from http://www.cardiogramplusc4d.org/data-downloads/ for coronary artery disease (CARDIoGRAM); and from Surendran et al., 2020 doi.org/10.1038/s41588-020-00713-x for hypertension.

Research involving human participants, their data, or biological material

Policy information about studies with human participants or human data. See also policy information about sex, gender (identity/presentation), and sexual orientation and race, ethnicity and racism.

Reporting on sex and gender	The genetic analyses included a sex covariate, and we used sex as self-reported by the UK Biobank participants or replication cohorts participants.
Reporting on race, ethnicity, or other socially relevant groupings	We used 'White British' and 'European' categories to refer genetically similar populations, as defined by principal component analyses. LURIC and ORCADES are reported as including German and Scottish participants, respectively.
Population characteristics	Analyses were adjusted for sex, supplement use, and environmental UVB radiation data.
Recruitment	Participants were recruited by the individual cohorts UK Biobank, LURIC, and ORCADES.
Ethics oversight	UK Biobank ethical approval was granted by the North West Multi-centre Research Ethics Committee. Ethics approval was similarly obtained by the original investigators for the replication cohorts.

Note that full information on the approval of the study protocol must also be provided in the manuscript.

Field-specific reporting

Please select the one below that is the best fit for your research. If you are not sure, read the appropriate sections before making your selection.

☒ Life sciences ☐ Behavioural & social sciences ☐ Ecological, evolutionary & environmental sciences

For a reference copy of the document with all sections, see nature.com/documents/nr-reporting-summary-flat.pdf

Life sciences study design

All studies must disclose on these points even when the disclosure is negative.

Sample size	Samples included any participants that passed quality control and had all relevant data available (i.e. genetic, vitamin D concentration, and covariate data). The discovery sample included all White British participants (N=338,977) and the replication sample included European participants (N=21,875), LURIC (N=2,909), ORCADES (N=1,875).
Data exclusions	Participants were excluded if they did have not genetic data, 25OHD measurement, or address data available. We only used participants of White British ancestry for the main analysis as this was the largest group in the UKB cohort and other subgroups were not sufficiently powered for the discovery analysis.
Replication	We used the European subgroup, LURIC, and ORCADES cohorts described above to perform replication analysis for the significant independent variants.
Randomization	Randomization is not relevant. For the subgroup analyses, participants were allocated to each subgroup based on their BMI category or UVB exposure level.
Blinding	Not applicable.

Reporting for specific materials, systems and methods

We require information from authors about some types of materials, experimental systems and methods used in many studies. Here, indicate whether each material, system or method listed is relevant to your study. If you are not sure if a list item applies to your research, read the appropriate section before selecting a response.

Materials & experimental systems

n/a	Involved in the study
☒	☐ Antibodies
☒	☐ Eukaryotic cell lines
☒	☐ Palaeontology and archaeology
☒	☐ Animals and other organisms
☒	☐ Clinical data
☒	☐ Dual use research of concern
☒	☐ Plants

Methods

n/a	Involved in the study
☒	☐ ChIP-seq
☒	☐ Flow cytometry
☒	☐ MRI-based neuroimaging

Plants

Seed stocks

Report on the source of all seed stocks or other plant material used. If applicable, state the seed stock centre and catalogue number. If plant specimens were collected from the field, describe the collection location, date and sampling procedures.

Novel plant genotypes

Describe the methods by which all novel plant genotypes were produced. This includes those generated by transgenic approaches, gene editing, chemical/radiation-based mutagenesis and hybridization. For transgenic lines, describe the transformation method, the number of independent lines analyzed and the generation upon which experiments were performed. For gene-edited lines, describe the editor used, the endogenous sequence targeted for editing, the targeting guide RNA sequence (if applicable) and how the editor was applied.

Authentication

Describe any authentication procedures for each seed stock used or novel genotype generated. Describe any experiments used to assess the effect of a mutation and, where applicable, how potential secondary effects (e.g. second site T-DNA insertions, mosiacism, off-target gene editing) were examined.
